# Taurine protects R28 cells from hypoxia/re-oxygenation-induced damage via regulation of mitochondrial energy metabolism

**DOI:** 10.1007/s00726-022-03199-5

**Published:** 2022-09-02

**Authors:** Wei Lu, Yuting Yang, Shunxiang Gao, Jihong Wu, Xinghuai Sun

**Affiliations:** 1 Department of Ophthalmology and Visual Science, Eye & ENT Hospital, Shanghai Medical College, Fudan University, Shanghai, 200031 China; 2 State Key Laboratory of Medical Neurobiology and MOE Frontiers Center for Brain Science, Institutes of Brain Science, Fudan University, Shanghai, 200032 China; 3 NHC Key Laboratory of Myopia, Chinese Academy of Medical Sciences, and Shanghai Key Laboratory of Visual Impairment and Restoration (Fudan University), Shanghai, 200031 China

**Keywords:** Taurine, GTPBP3, Mitochondrial energy metabolism, Seahorse, Hypoxia/re-oxygenation

## Abstract

Oxidative-induced damage and hypoxia/re-oxygenation (H/R) injury are common causes of irreversible visual impairment. The goals of this study were to explore the effects of taurine on R28 cells under the two damage models and the underlying mechanisms. Low doses of taurine supplementation promoted cell viability, mitochondrial membrane potential (MMP), SOD levels, ATP contents and attenuated cytotoxicity and intracellular ROS generation of the R28 cells under the two kinds of damage. The expression level of GTPBP3, a mitochondrial-tRNA (mt-tRNA) modification enzyme that catalyzes the taurine involved modification, was decreased under the two damage and taurine could reverse the reduction. After knocking down GTPBP3, the R28 cells become vulnerable to damage. The viability, cytotoxicity, MMP and intracellular ROS level of knockdown cells changed more obviously under the H/R injury than those of control cell. We also found that knockdown of GTPBP3 significantly decreased mitochondrial energy metabolism by measuring the oxidative respiration rate by the Seahorse XFe24 extracellular flux analyzer. The protection of low doses of taurine disappeared on knockdown R28 cells, indicating that GTPBP3 is crucial in the protection mechanisms of taurine. However, the impacts of the reduction of GTPBP3 level can be reversed by relatively high doses of taurine, implying the protection effects of taurine were dose-dependent, and there were more complicated mechanisms remain to be explored. This study explored a new mechanism of the neuroprotective effects of taurine, which depend on the GTPBP3-mediated taurine modification of mt-tRNAs and the promotion of mitochondrial energy metabolism.

**Supplementary Information:**

The online version contains supplementary material available at 10.1007/s00726-022-03199-5.

## Introduction

Glaucoma is one of the leading causes of blindness worldwide and elevated intraocular pressure (IOP) is a major risk factor for retinal ganglion cells (RGC) death and visual field defects (Sun et al. 2017). IOP fluctuation influences retinal blood flow and causes retinal hypoxia/re-oxygenation (H/R) injury, a common cause of irreversible visual impairment (Osborne et al. 2004; Grieshaber and Flammer 2005). Restoration of blood circulation causes inflammation and oxidative stress-induced damage in the area affected by the absence of oxygen during retinal ischemia. Extensive research has shown that mitochondrial damage, particularly impaired mitochondrial oxidative metabolism, is the main cause of cellular injury and organ failure during H/R stress (Kalogeris et al. 2012). Appropriate methods should be adopted to limit the degree of H/R damage and enhance the resistance to cell death.

Taurine is a non-protein amino acid involved in a wide range of physiological processes (Ripps and Shen 2012). Recently, in in vivo and in vitro, taurine has been demonstrated to exert neuroprotective effects and various therapeutic properties against various disorders through the regulation of osmolarity, antioxidant properties, calcium modulation and activation of inhibitory neurotransmitters (Jakaria et al. 2019; Wu and Prentice 2010). Several studies have shown that taurine protects against *N*-methyl-d-aspartate and endothelin-1 induced retinal damage, as well as RGC loss in glaucomatous animal models by regulating apoptosis and oxidative stress (Jafri et al. 2019; Nor Arfuzir et al. 2018; Froger et al. 2012). Additionally, taurine depletion has been demonstrated to diminish the thickness of the retinal nerve fiber layer and damage RGC, which can be alleviated by taurine supplementation (Garcia-Ayuso et al. 2019).

Mitochondria contain their own genome, which encodes 13 essential subunits of the oxidative respiratory chain (ORC) complexes (7 of complex I, 1 of complex III, 3 of complex IV and 2 of complex V) (Suzuki et al. 2011). Following transcription, mitochondrial tRNAs (mt-tRNAs) are modified by nuclear-encoded tRNA-modifying enzymes. These modifications are required for the proper functioning of mt-tRNAs, and the absence of these modifications can influence the translation of ORC subunits, affecting the function of ORC and the efficiency of oxidative phosphorylation. Taurine participates in the formation of 5-taurinemethyluridine (τm^5^U) and 5-taurine-methyl 2-thiouridine (τm^5^s^2^U), which are present at wobble position U34, the first nucleotide of the anticodon (Suzuki et al. 2011). The specific taurine-linked post-transcriptional modification of the wobble base is one of the few modification types that exist only in mt-tRNAs, not in cytosolic tRNAs (Suzuki and Suzuki 2014). This modification enhances the correct folding and stability of certain mt-tRNAs, improving the binding of the anticodon to the codon and enhancing mitochondrial translation efficiency.

Based on genetic studies, researchers have predicted that GTP-binding protein 3 (GTPBP3) is likely to be the mt-tRNA-modifying enzyme responsible for taurine modification and involved in the formation of the τm^5^U (Asano et al. 2018; de Crecy-Lagard et al. 2019). Researchers have reported that patients carrying multiple mutations of *GTPBP3* presented with combined respiratory chain complex deficiencies in skeletal muscle, as well as visual impairment (Kopajtich et al. 2014). Whole exome sequencing results of a primary angle closure glaucoma (PACG)-enriched family indicated a higher variant frequency of a single-nucleotide variant of *GTPBP3* than that in control samples, demonstrating that glaucoma should be one among the oxidative phosphorylation diseases and may be affected by GTPBP3 (Qiao et al. 2020).

To explore the protective effects of taurine on glaucomatous injury and the underlying mechanisms associated with GTPBP3 and mitochondrial, we used the immortalized retinal progenitor cell line R28, a hydrogen peroxide (H_2_O_2_)-induced oxidative stress model, and H/R model to mimic the conditions for glaucomatous injury in vitro. Our results provide novel insights into the neuroprotective properties of taurine, which involves the regulation of mitochondrial energy metabolism by influencing ORC function through GTPBP3.

## Materials and methods

### Cell culture

The R28 cell line was generously provided by Dr. Guotong Xu (Tongji Eye Institute, Tongji University School of Medicine, Shanghai, China) and grown in low-glucose DMEM (Sigma-Aldrich, MO, USA) supplemented with 10% fetal bovine serum (Thermo Fisher Scientific, CA, USA) and 1% penicillin/streptomycin (Thermo Fisher Scientific, CA, USA) in a humidified incubator at 37 °C with 5% CO_2_. For inducing oxidative stress, R28 cells were seeded for 24 h and then treated with different concentrations of taurine (T8691, Sigma-Aldrich, MO, USA) (0.1, 1, 5, 10 or 20 mM) or isometric PBS for 4 h before treatment with 250 μM H_2_O_2_ for 24 h. To induce H/R injury in vitro, we cultured the R28 cells under a normoxic condition (95% air, 5% CO_2_) for 24 h and then treated with different concentrations of taurine (0.1, 1, 5, 10 or 20 mM) or isometric PBS for 4 h before being placed under a hypoxic condition (0.2% O2, 5% CO_2_) at 37 °C for 24 h in a triple gas incubator. The cells were then cultured under the normoxic condition for another 24 h. Cells incubated in complete medium under the normoxic condition were used as the control.

### Cell viability and cytotoxicity

R28 cells were seeded in 96-well plates (2 × 10^4^ cells/well), and treated in various ways. Cell viability was assayed using the Cell Counting Kit-8 (CCK-8) (Beyotime Biotechnology, Shanghai, China). In brief, 10 μL CCK-8 was added to each sample for 2 h at 37 °C and the absorbance at 450 nm was measured by a microplate reader. Cytotoxicity was determined using Cytotoxicity Lactate Dehydrogenase (LDH) assay kit (Dojindo, Kumamoto, Japan) according to the manufacturer's guidelines. Briefly, after adding 100 μL working solution per well, the plates were cultured at room temperature for 10 min away from light and absorbance was measured at 490 nm by a microplate reader. Viability and cytotoxicity are presented as percentage relative to the values in the control groups.

### Detection of superoxide dismutase (SOD)

R28 cells were evenly planted in six-well plates (5 × 10^5^ cells/well) and treated in various ways. SOD activity was assayed using the SOD assay kit-WST-8 method (Beyotime Biotechnology, Shanghai, China) according to the manufacturer's instructions. Briefly, after lysis, samples were collected and centrifuged at 12,000 rpm for 5 min. The supernatant was collected and total protein was measured using Pierce™ bicinchoninic acid (BCA) protein assay kit (Thermo Fisher Scientific, CA, USA). According to the protein concentration, the samples were diluted with SOD assay buffer to 2 μg/μL. Next, 20 μL of each sample was added to a 96-well plate, followed by the WST-8/enzyme working solution, SOD assay buffer and reaction start working solution. After culturing at 37 °C for 30 min, the absorbance was measured at 450 nm using a microplate reader. Total SOD activity was calculated according to the manufacturer's instructions.

### Analysis of mitochondrial membrane potential (MMP)

R28 cells were evenly seeded in 96-well plates (2 × 10^4^ cells/well) and treated in various ways. Changes in MMP were explored using the JC-1 MMP assay kit (Beyotime Biotechnology, Shanghai, China) according to the manufacturer’s protocol. Briefly, the cells were incubated at 37 °C for 20 min with JC-1 working solution and washed three times with wash buffer; next, fresh medium was added. The fluorescence intensities of JC-1 monomers (excitation at 490 nm, emission at 530 nm) and aggregates (excitation at 525 nm, emission at 590 nm) were detected using a microplate reader. MMP was determined as the ratio of the fluorescence intensity of JC-1 aggregates and monomers.

### Detection of reactive oxygen species (ROS)

The intracellular ROS levels were measured using a ROS Assay Kit (Beyotime Biotechnology, Shanghai, China). Briefly, R28 cells were evenly planted in 96-well plates (2 × 10^4^ cells/well) and treated in different ways. Following treatments, the cells were incubated with 2′,7′-dichlorofluorescein-diacetate (DCFH-DA), which is easily oxidized to fluorescent dichlorofluorescein (DCF) by intracellular ROS, for 20 min at 37 °C and then washed twice with PBS. The fluorescence intensity was observed by a fluorescence microscope (Carl Zeiss, Germany) and the average fluorescence intensity was analyzed by the Image J software (National Institutes of Health, USA).

### Detection of adenosine triphosphate (ATP)

ATP content in R28 cells was detected using an Enhanced ATP assay kit (Beyotime Biotechnology, Shanghai, China) according to the manufacturer’s protocol. Briefly, after lysis, samples were collected and centrifuged at 12,000 rpm at 4 ℃ for 5 min. The supernatant was collected. ATP working solution (100 μL) was added to the detection well in a white 96-well plate for 5 min at room temperature. Isometric samples and diluted ATP standard solution (0.01, 0.05, 0.1, 0.5, 1, 5, and 10 μM) were added to the detection wells and tested using a luminometer program of a microplate reader. The ATP levels were calculated according to the standard curve and normalized to the amount of protein in each sample after protein quantification using BCA protein assay kit.

### Measurement of intracellular taurine level

Intracellular taurine levels were measured using gas chromatography–mass spectrometry (GC-TOFMS, Leco Corp, MI, USA). R28 cells were dissolved in 225 μL methanol and centrifuged at 18,000 rpm at 4 ℃ for 25 min. Internal standard with a volume of 10 μL was added to 160 μL supernatant solution and then lyophilized. Next, 50 μL methoxyamine solution (15 mg/mL in pyridine) and 50 μL MSTFA were added to the samples in sequence and reacted at 37 °C for 2 and 1 h, respectively, and were ready for analysis. The value of taurine was expressed as nmol per million cells.

### Real-time quantitative polymerase chain reaction (qRT-PCR)

EZ-press RNA Purification Kit (EZBioscience, Shanghai, China) was used to isolate total RNA from R28 cells according to the instructions provided by the manufacturer. Reverse Transcription Master Mix (EZBioscience, Shanghai, China) were utilized for cDNA synthesis. qRT-PCR was performed on PCR System (Bio-Rad Real-Time; Bio-Rad Laboratories, Hercules, CA, USA) with SYBR Green qPCR Master Mix (EZBioscience, Shanghai, China) using the following primers: Actin: forward 5′-CACCCGCGAGTACAACCTTC-3′, reverse 5′-CCCATACCCACCATCACACC-3′; GTPBP3: forward 5′-GACTTCGGAGAGGATGATAA-3′, reverse 5′-AATGGACACTGGCTTCTG-3′.

### Western blotting

R28 cells were lysed in Western and Immunoprecipitation Lysis Buffer (Beyotime Biotechnology, China) with Protease Inhibitor Cocktail (Abcam, UK). After incubated on ice for 5 min, the samples were collected and centrifuged at 12,000 rpm for 15 min. The total amount of protein was determined by Pierce™ BCA protein assay kit (Thermo Fisher Scientific, CA, USA). Proteins were electrophoresed on SurePAGE Gels (GenScrip, Shanghai, China) and transferred to PVDF membranes (Millipore, MA, USA). The membranes were incubated with anti-GTPBP3 (1:2000, Thermo Fisher Scientific, CA, USA) and anti-β-Actin (1:5000, Proteintech, Shanghai, China) overnight at 4 °C. After washing with TBST for three times, the membranes were incubated with secondary antibodies for 1 h at room temperature. The gray densities of GTPBP3 bands were normalized to the densities of β-actin bands with Image J software (National Institutes of Health, USA).

### Lentiviral transfection

Short hairpin RNA (shRNA) targeting GTPBP3 was designed and constructed by Hanheng Biotechnology (Shanghai, China). The following sequences were inserted into a lentiviral shRNA vector: forward: 5′-GATCCGACATTGACTTCGGAGAGGATGATAATTCAAGAGATTATCATCCTCTCCGAAGTCAATGTTTTTTTG-3′, reverse: 5′-AATTCAAAAAAACATTGACTTCGGAGAGGATGATAATCTCTTGAATTATCATCCTCTCCGAAGTCAATGTCG-3′. A negative control shRNA directed to the lentiviral vector was generated using 5′-GATCCGTTCTCCGAACGTGTCACGTAATTCAAGAGATTACGTGACACGTTCGGAGAATTTTTTC-3′ (forward) and 5′-AATTGAAAAAATTCTCCGAACGTGTCACGTAATCTCTTGAATTACGTGACACGTTCGGAGAACG-3′ (reverse). R28 cells were cultured until confluence reached approximately 30% and then transfected with lentiviral vectors (titer: 1 × 10^9^) containing GTPBP3 shRNA (KD group) or negative control shRNA (NC group) at a multiplicity of an infection ratio of 20. In brief, 2 × 10^5^ R28 cells were incubated with 40 μL lentivirus, 8 µg polybrene (Hanheng Biotechnology, Shanghai, China) and 1 mL culture medium in a 6-well plate at 37 °C with 5% CO_2_ for 4 h. Next, 1 mL fresh growth medium was added per well and the culture medium was replaced with fresh growth medium 24 h later. Puromycin (2 μg/mL) was added to the medium 48 h after transfection to generate stably transfected cells.

### Mitochondrial staining with mito-tracker (mitochondrion-selective probes) and immunocytochemistry

Cells transfected with lentivirus grown on coverslips and incubated in medium containing 200 nM Mitotracker Red CMXRos (Thermo Fisher Scientific, CA, USA) at 37 °C for 30 min. Subsequently, the cells were washed three times with PBS and then fixed with 4% PFA for 15 min at room temperature, followed by incubation in PBS containing 0.5% Triton X-100 and 5% goat serum (Beyotime Biotechnology, Shanghai, China) for 60 min to permeate and block nonspecific binding. The cells were then incubated with primary antibodies against GTPBP3 (1:100; Thermo Fisher Scientific, CA, USA) overnight at 4 ℃. After washing three times with PBS, the cells were incubated with Alexa Fluor 488 goat anti-rabbit (1:500, Thermo Fisher Scientific, CA, USA) and mounted with mounting medium with DAPI (Sigma-Aldrich, MO, USA). All the procedures above should be protected from light. Images were captured with a laser confocal microscope (Leica SP8, Wetzlar, German) and processed using Image J (National Institutes of Health, USA). Co-localization of the mitochondria and GTPBP3 was calculated as Manders’ Co-localization Coefficient (MCC) using Coloc 2 plugin.

### Measurement of mitochondrial oxygen consumption rate (OCR)

The OCR of R28 cells transfected with or without lentivirus was measured using an extracellular flux analyzer XF24 (Seahorse Bioscience, MA, USA). Cells were plated in a Seahorse 24-well microplate (Seahorse Bioscience, MA, USA) (2 × 10^4^ cells/well) and cultured in complete DMEM growth medium for 24 h in a 5% CO_2_ incubator at 37 °C. Next, the medium was removed and cells were washed by XF assay medium (Seahorse Bioscience, MA, USA) twice and cultured in the XF assay medium for 1 h at 37 °C to allow temperature and pH to reach equilibrium. Mitochondrial complex inhibitors (oligomycin, FCCP and antimycin A/rotenone) were freshly prepared in the XF assay medium. After 26 min of measuring basal respiration, oligomycin (1.5 μM), which inhibits ATP synthase, was injected through reagent delivery chambers in each well of the microplate to measure basal mitochondrial ATP synthesis. FCCP (1 μM), which uncouples the mitochondria, was injected at 50 min to obtain the maximum OCR. Finally, a mixture of antimycin A (0.5 μM), an electron transport blocker, and rotenone (0.5 μM), an inhibitor of mitochondrial complex I, was injected at 74 min to shut down mitochondrial respiration and confirm that the respiration changes were mainly due to the mitochondrial. Upon completion of the assay, Hoechst 33342 and propidium iodide were used to stain living and dead cells, respectively. The cell numbers were automatically counted using Ensight (PerkinElmer, MA, USA). The OCR was recorded as pmol/ min and normalized to the total number of cells in each well. The averages of three or four wells were calculated for each data point.

### Statistical analysis

All results are reported as the mean ± standard error of the mean (SEM) obtained from at least three independent experiments. The statistical significance of the data was determined using Student’s t test for comparisons between groups and one-way analysis of variance (ANOVA) and Dunnett's multiple comparisons test for comparisons among groups. Statistical significance was set at P < 0.05. Statistical analyses were performed using GraphPad Prism software (version 7.0; La Jolla, CA, USA).

## Results

### Taurine protects R28 cells against H_2_O_2_-induced damage and H/R-induced damage

To investigate whether taurine influences R28 cells under normal conditions, we treated R28 cells with 0.1, 1, and 5 mM taurine for 24 h and measured their viability, cytotoxicity and MMP. The results showed that 0.1, 1, and 5 mM taurine did not significantly affect the proliferation of R28 cells (Supplementary Fig. 1A). However, 5 mM taurine significantly reduced cytotoxicity and increased MMP of R28 cells (Supplementary Fig. 1B, C). We detected the intracellular taurine in R28 cells at the basal level, which was 3 nmol/million cells. H_2_O_2_ did not cause a significant reduction in taurine content in R28 dells (Supplementary Fig. 2).

**Fig. 1:**
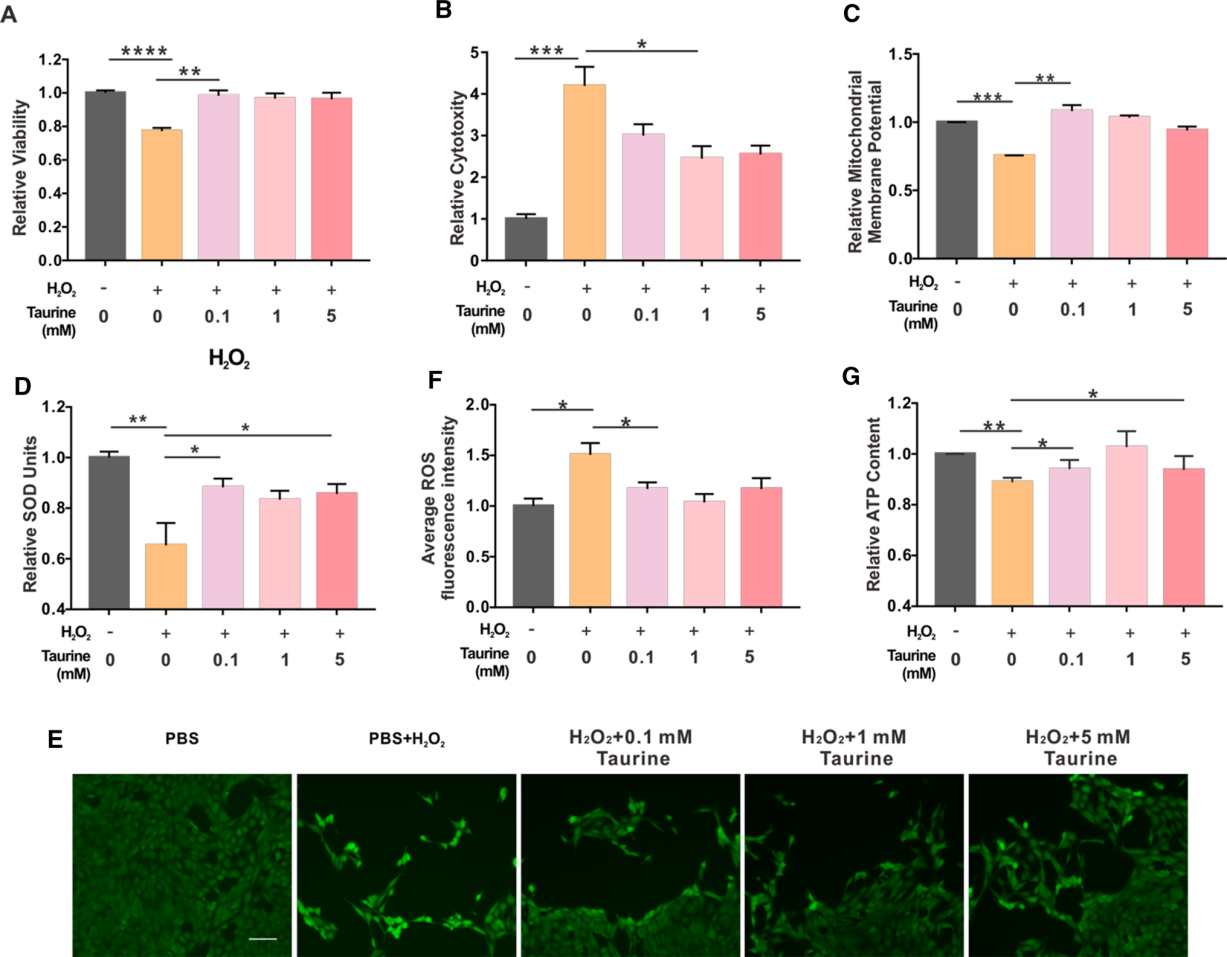
Taurine protects R28 cells against H_2_O_2_-induced damage. **A**–**D** CCK-8, LDH, MMP and SOD level changes in R28 cells under H_2_O_2_-induced oxidative stress with or without taurine administration, **E** representative images of ROS level under different conditions, **F** average fluorescence intensity of ROS images analyzed by Image J software. **G** ATP contents of R28 cells under different conditions. *p < 0.05, **p < 0.01, ***p < 0.001, ****p < 0.0001. *Scale bar*: 100 μm

To investigate whether taurine could protect R28 cells against H_2_O_2_-induced damage, we treated R28 cells with 0.1, 1, and 5 mM taurine for 4 h before H_2_O_2_ was added to the culture medium. After 24 h of treatment, the CCK-8 and LDH assays were performed. The results showed that the proliferation and death of R28 cells treated with only H_2_O_2_ were significantly decreased and increased, respectively, and taurine alleviated these effects (Fig. 1A, B). The MMP of the H_2_O_2_ group decreased to approximately 75% of non-treated control cells, and 0.1 mM taurine increased it to 86% (Fig. 1C). Several researchers have demonstrated that the potent neuroprotective properties of taurine are associated with its antioxidant capacity; therefore, we explored the effects of taurine on H_2_O_2_-induced oxidative stress by measuring SOD activity and ROS levels. The SOD level of the H_2_O_2_ group was approximately 65% of that of the control group, and 0.1 mM taurine enhanced the SOD level to 88% (Fig. 1D). A significant increase in mitochondrial ROS levels relative to the control group was observed in the analysis of the fluorescence intensity of ROS (Fig. 1E, F). The administration of taurine reversed these impacts (Fig. 1E, F). ATP content of R28 cells under H_2_O_2_-induced oxidative stress was also measured. The ATP level in the H_2_O_2_ group decreased to 88% of the control group. Administration of taurine reversed this effect (Fig. 1G). Moreover, 0.1, 1, and 5 mM taurine tended to increase intracellular taurine levels in H_2_O_2_-induced damage (Supplementary Fig. 2). These results demonstrate the protective effects of taurine against H_2_O_2_-induced damage in R28 cells.

**Fig. 2:**
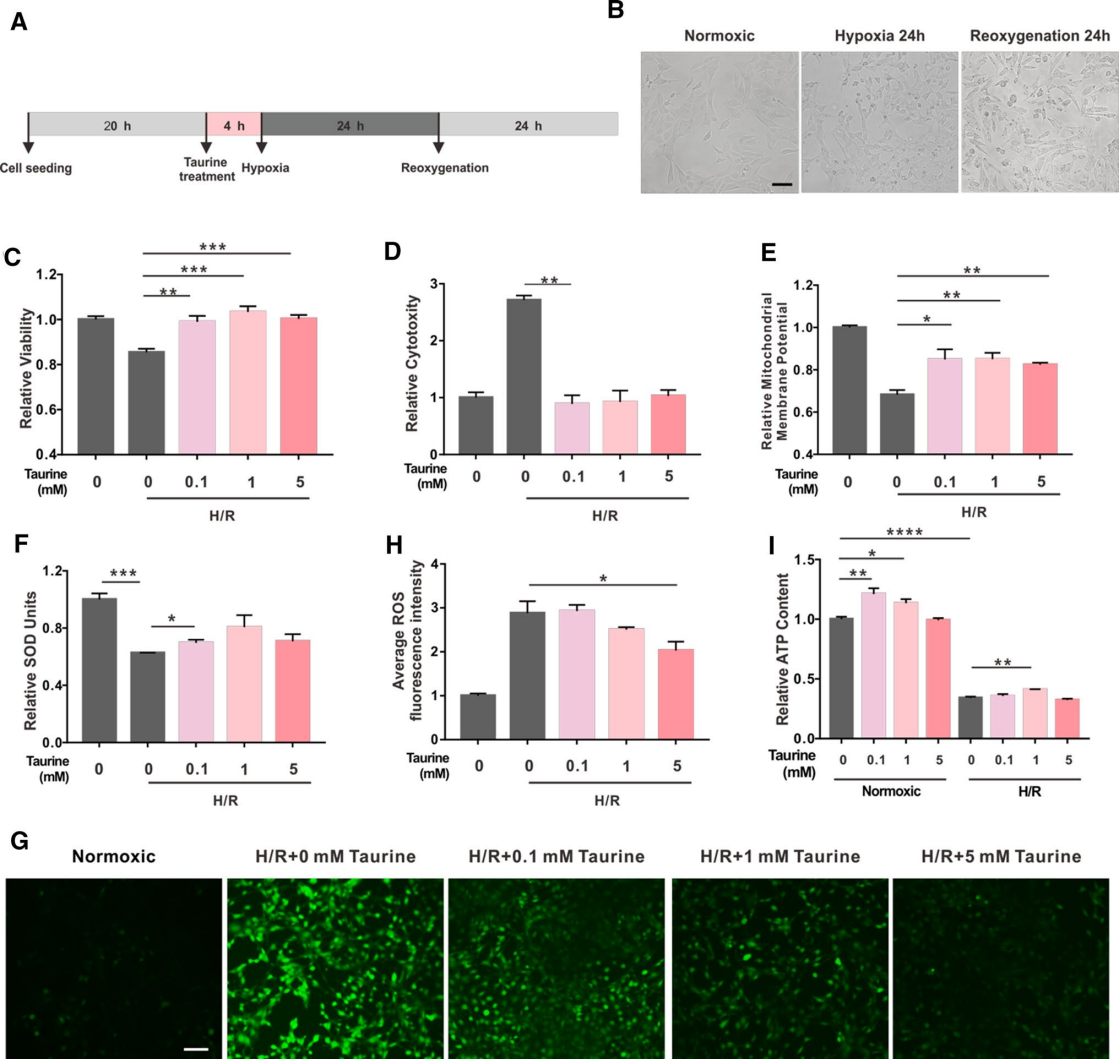
Taurine protects R28 cells against H/R-induced damage. **A** Experimental design of H/R-induced damage, **B** light field images of R28 cells during H/R injury, **C**–**F** CCK-8 results, LDH results, MMP and SOD level changes in R28 cells under H/R-induced damage with or without taurine treatment, **G** representative images of ROS level under different conditions, **H** average fluorescence intensity of ROS images analyzed by Image J software. **I** ATP content of R28 cells under different conditions. *p < 0.05, **p < 0.01, ***p < 0.001. *Scale bar*: 100 μm

To further verify the protective role of taurine, we investigated its neuroprotective effects on R28 cells after H/R injury (Fig. 2A). Figure 2B shows light field images of R28 cells during H/R injury. After 24 h of hypoxia, R28 cells became shrunken and rounded. This morphological damage increased in severity after 24 h of re-oxygenation. As shown in Fig. 2C, the average proliferation rate of R28 cells decreased by 15% after H/R injury. Remarkably, 0.1, 1, and 5 mM taurine promoted cell viability to a level close to that of the control group (Fig. 2C). Cytotoxicity of R28 cells under H/R conditions increased 2.7-fold and was reversed to near-normal levels by taurine (Fig. 2D). A significant decrease in the MMP (Fig. 2E), a decrease in SOD levels (Fig. 2F), and an increase in ROS levels (Fig. 2G, H) relative to the control groups were observed, and these changes were significantly reversed by the administration of taurine. Notably, taurine significantly increased the ATP content in R28 cells under normoxic conditions (Fig. 2I). Under H/R damage, the ATP level decreased to 34% of normoxic control group and could be significantly increased to 40% by 1 mM taurine (Fig. 2I). Thus, these results demonstrate the protective effects of taurine against H/R-induced damage.

### Taurine modulates expression of GTPBP3 in both H_2_O_2_-induced damage and H/R-induced damage

**Fig. 3:**
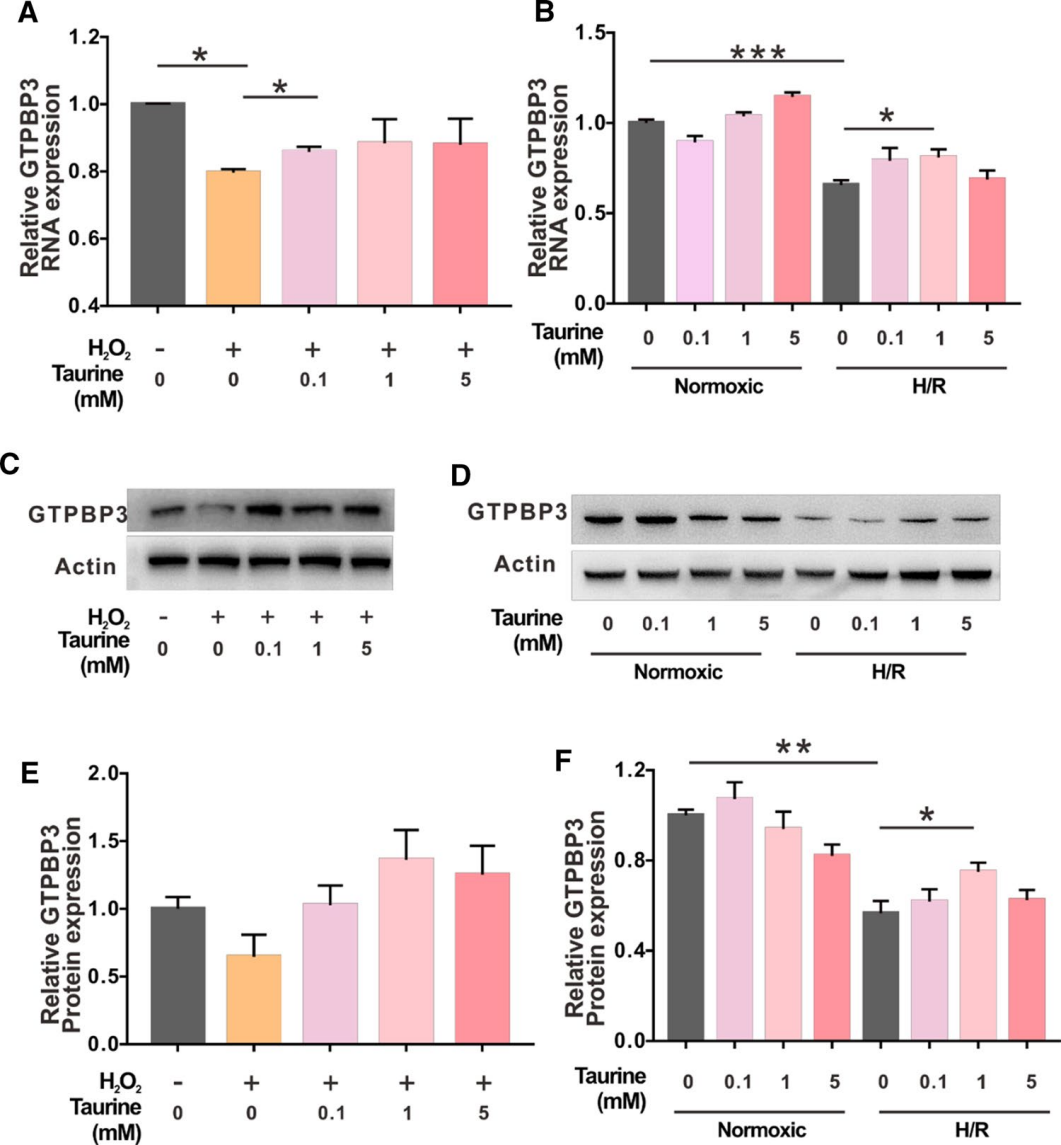
Expression level of GTPBP3 in R28 cells under the two types of damage. **A**, **B** mRNA expression level of GTPBP3 in R28 cells under H_2_O_2_-induced damage, normal condition, and H/R-induced damage, **C**, **D** immunoblotting of GTPBP3 and ACTIN under the two types of damage, E, **F** protein levels of GTPBP3 were quantified by Image J software. *p < 0.05, **p < 0.01, ***p < 0.001

To explore whether taurine influences the expression of GTPBP3, we examined GTPBP3 at the RNA and protein levels in the two types of damage models. Under normal conditions, 5 mM taurine up-regulated GTPBP3 mRNA expression (Fig. 3B). The mRNA expression of GTPBP3 significantly decreased to 79% and 65% during H_2_O_2_-induced damage and H/R-induced damage, respectively. Administration of taurine attenuated these effects in both models (Fig. 3A, B). The protein expression of GTPBP3 also decreased compared with that in the control groups during both types of damage. Similar to the effects on RNA, the administration of taurine enhanced GTPBP3 protein levels (Fig. 3C, F). These results indicate that the expression level of GTPBP3 changes significantly under different damage conditions and can be regulated by taurine, suggesting that taurine may indirectly promote mt-tRNA modification and ORC function by regulating the expression of GTPBP3 under damage conditions.

### Knockdown of *GTPBP3* influences the proliferation and mitochondrial respiration of R28 cells

**Fig. 4:**
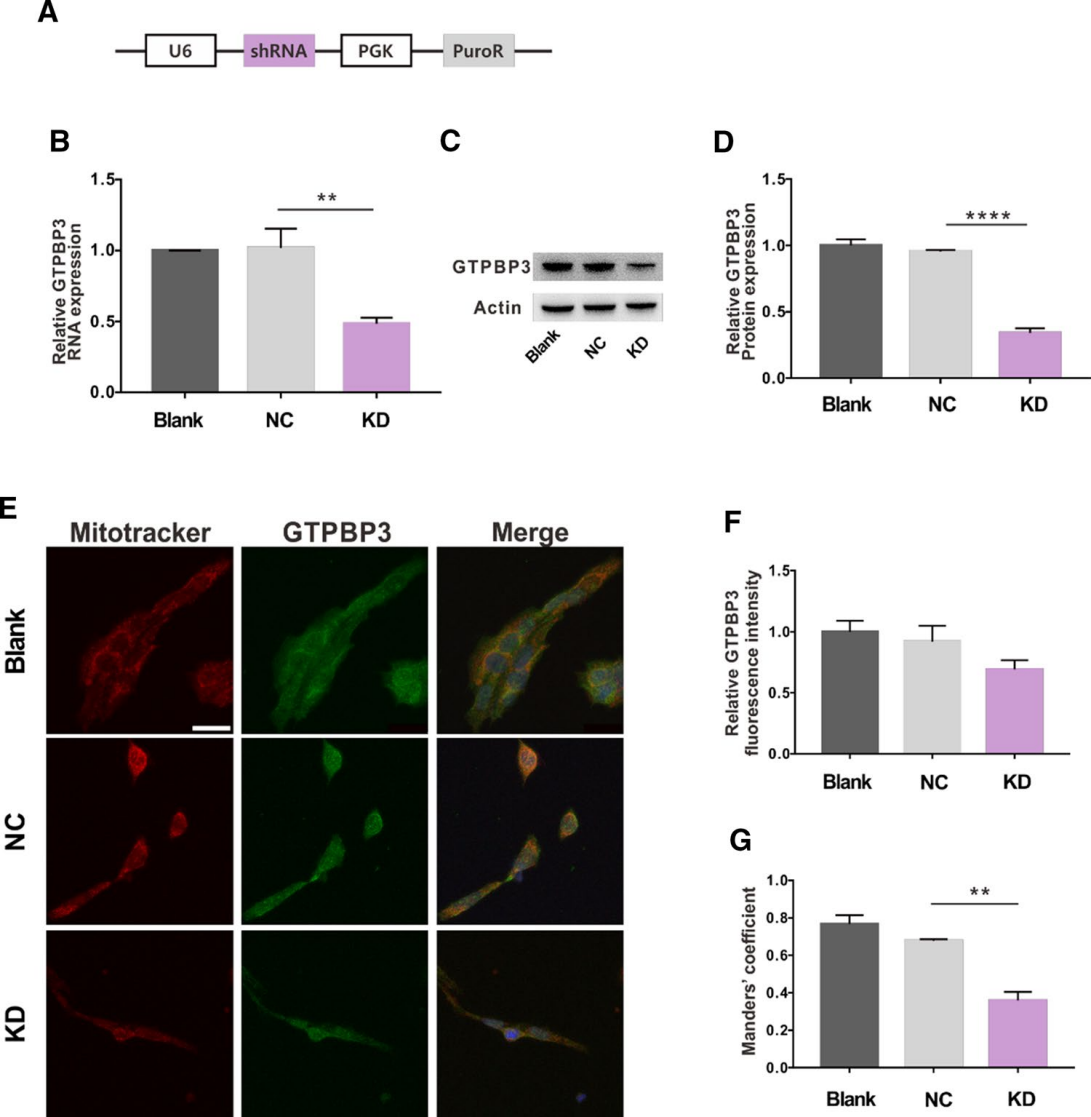
Expression level changes in GTPBP3 in R28 cells with lentiviral transfection. **A** Main functional elements in lentiviral vectors. Promotor, U6 and PGK; selection marker, puromycin; **B** mRNA expression level, **C** immunoblotting of GTPBP3 and ACTIN, **D** protein levels of GTPBP3 were quantified by Image J, **E** representative images of the staining of Mito-Tracker and GTPBP3 in KD group and control groups, **F** analysis of average fluorescence intensity of GTPBP3, **G** analysis of co-localization of Mito-Tracker and GTPBP3. **p < 0.01, ****p < 0.0001. *Scale bar*: 20 μm

**Fig. 5:**
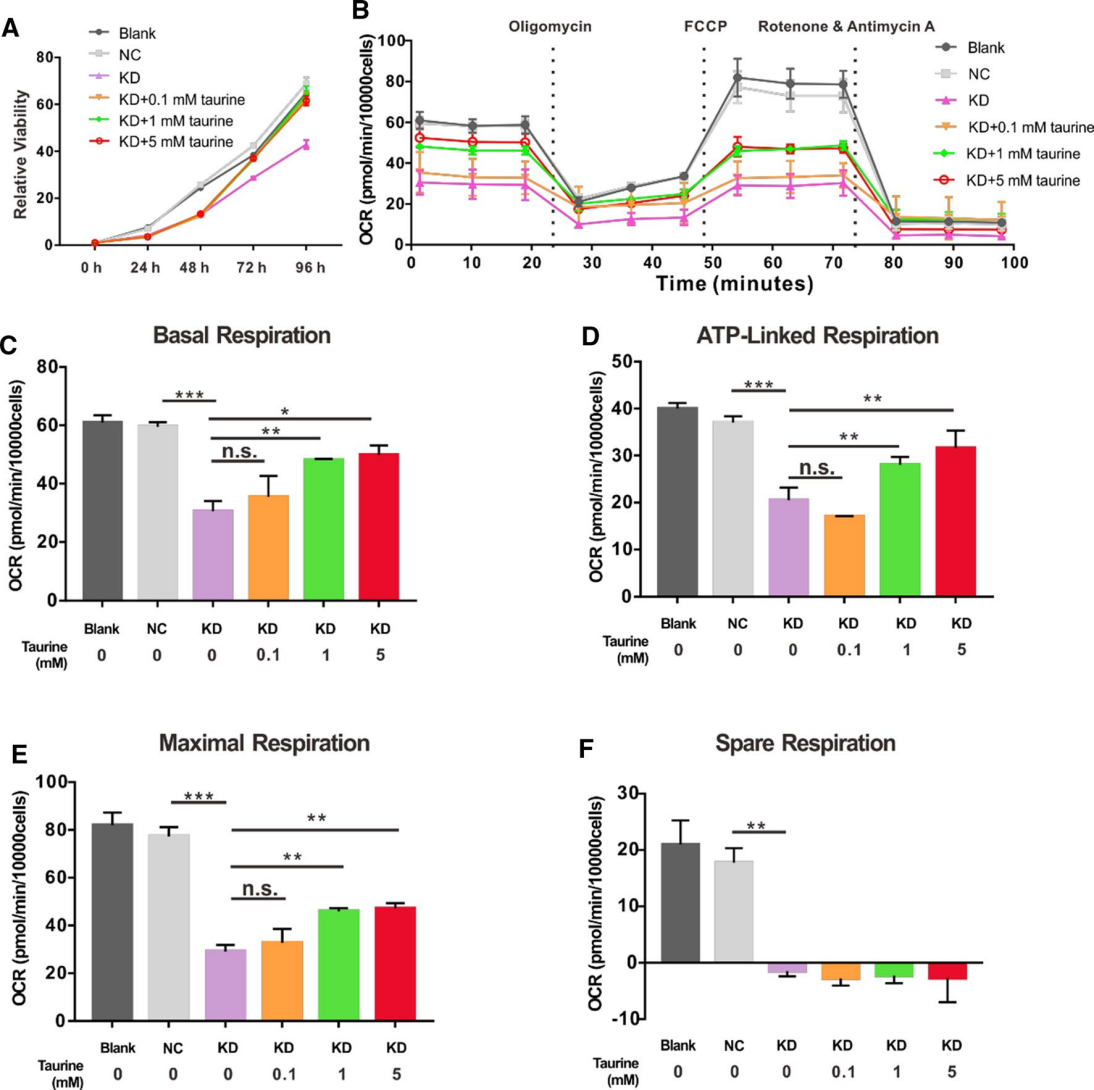
Reduction of GTPBP3 influences proliferation and mitochondrial respiration of R28 cells. **A** Proliferation rate of control groups and KD group with or without taurine treatment, **B** OCR at baseline and after sequential injection of the three drugs under different conditions, **C**–**F** analysis of basal respiration, ATP-linked respiration, maximal respiration, and spare respiratory capacity. *p < 0.05, **p < 0.01, ***p < 0.001, n.s., not significant

**Fig. 6:**
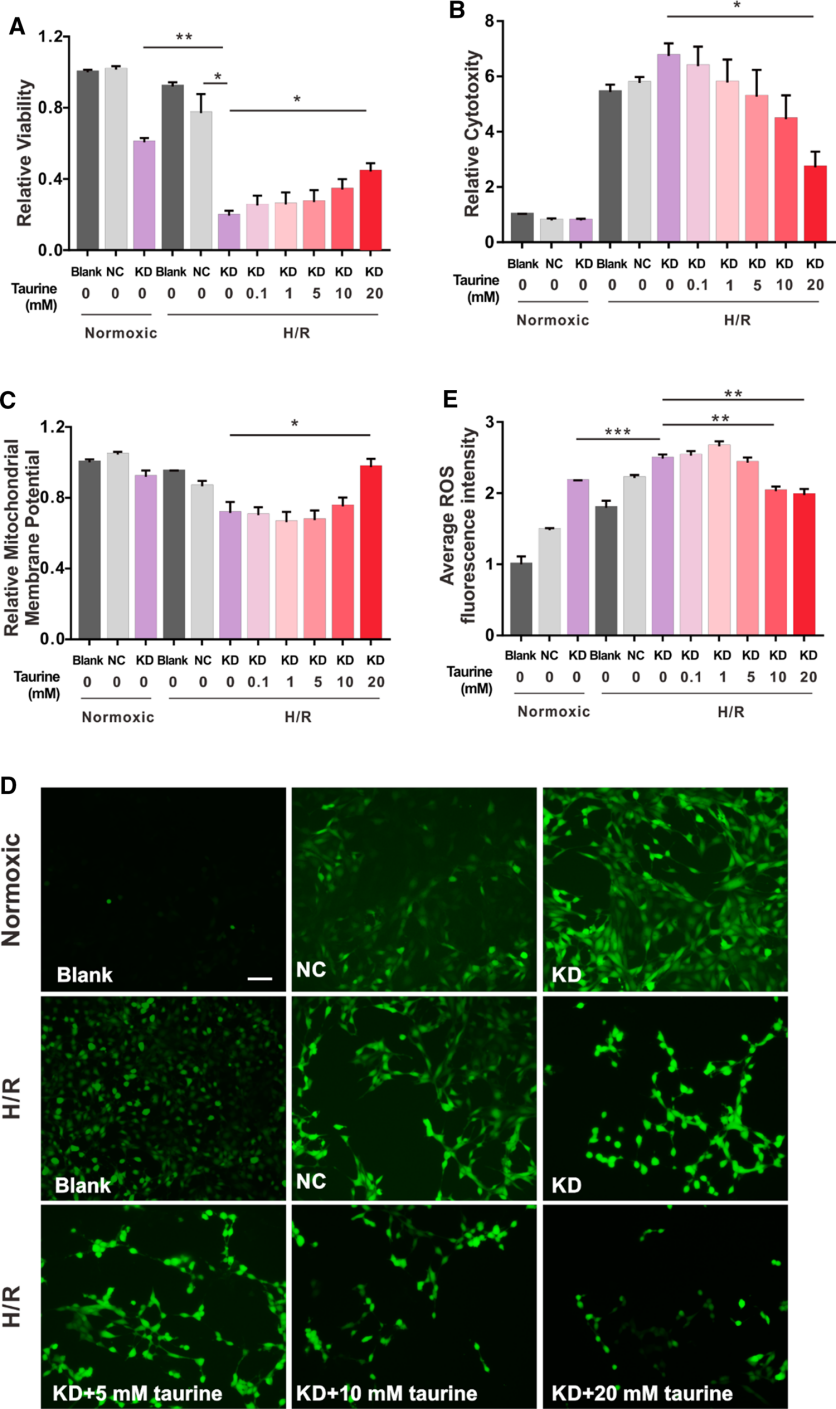
Reduced GTPBP3 expression increased the vulnerability of R28 cells; low taurine concentrations cannot reverse the effects, but higher concentrations can. **A**–**C** CCK-8 results, LDH results and MMP changes in NC group and KD group under H/R or normoxic condition, with or without taurine treatment, **D** representative images of ROS level under different conditions, **E** average fluorescence intensity of ROS images analyzed by Image J software. *p < 0.05, **p < 0.01, ***p < 0.001. *Scale bar*: 100 μm

To elucidate the significance of GTPBP3 in R28 cells, we used a lentiviral shRNA vector to knock down *GTPBP3* and explore its effects (Fig. 4A). The mRNA and protein levels of GTPBP3 significantly decreased (Fig. 4B–D). Because GTPBP3 is synthesized in the cytoplasm and translocated to the mitochondria to catalyze mt-tRNA modification, we used immunocytochemistry and Mito-Tracker to observe changes in the expression and location of GTPBP3 in R28 cells. As shown in Fig. 4E, F, GTPBP3 expression was decreased in the knockdown cells. The co-localization of GTPBP3 and Mito-Tracker in the blank and NC groups was similar and significantly higher than that in the KD group (Fig. 4G), which indicates that functional GTPBP3 is also decreased. We also found that the cells in the KD group grew slower than those in the NC group (Figs. 5A and 6A). The proliferation rate of the KD group was not enhanced until 72 h after taurine treatments (Fig. 5A). There were no obvious changes in cytotoxicity, and MMP and ROS levels slightly decreased and increased in the KD group, respectively (Fig. 6B–E).

Because GTPBP3 catalyzes the modification of mt-tRNA and promotes ORC function, we measured mitochondrial respiration using the Seahorse XFe24 extracellular flux analyzer to further explore the influence of GTPBP3 reduction. As shown in Fig. 5B, C, knockdown of GTPBP3 in R28 cells caused a 50% decline in basal respiration compared with that in the control. Oligomycin, a complex V inhibitor, was the first to be injected into the assay, to determine the proportion of ATP-linked OCR in basal respiration. The next compound injected was FCCP, an uncoupling agent-used to determine the maximal respiration and calculate the spare respiratory capacity. ATP-linked respiration and maximal respiration in the KD group were reduced to ~ 54% and ~ 37%, respectively, compared with that in the NC group, and the spare respiratory capacity of the KD group was completely lost (Fig. 5D–F). These results indicate that the reduction in the expression of GTPBP3 significantly damaged mitochondrial energy metabolism in R28 cells.

### KD group cells are more vulnerable to H/R-induced damage than R28 cells, and 0.1, 1, and 5 mM taurine cannot reverse these effects

To test whether reduced GTPBP3 expression increases the vulnerability of R28 cells and whether taurine protects KD group from damage, we tested the effects of 0.1, 1, and 5 mM taurine on KD group cells under H/R damage. Compared with those under normoxic conditions, the viability and MMP of the NC group decreased to 76% and 82%, respectively, and cytotoxicity increased 7.7-fold under H/R conditions. The changes in the three parameters were more obvious in the KD group (31%, 77%, and 8-fold, respectively) than those in NC group. Although under H/R condition, the fold change in ROS level in the KD group was slightly smaller than that in the NC group (1.2-fold and 1.5-fold, respectively), the ROS level in the KD group under normoxic conditions was already significantly higher than that in the NC group. However, administration of 0.1, 1, and 5 mM taurine did not significantly reverse the effects of H/R injury (Fig. 6A–E). We also assessed the respiratory chain activity by measuring the OCR of taurine-treated knockdown cells. As shown in Fig. 5B–F, 0.1 mM taurine did not significantly affect the OCR of the KD group, and 1 and 5 mM taurine slightly enhanced basal respiration, ATP production-linked OCR, and maximal respiration. However, the spare respiratory capacity was indistinguishable from that of the untreated knockdown cells, which were completely lost. These results indicate that GTPBP3 is crucial for physiological function of R28 cells and that the protective effects of 0.1, 1, and 5 mM taurine are limited when the basal level of GTPBP3 is decreased.

### High concentrations of taurine alleviate H/R-induced damage and improve mitochondrial metabolism of KD group cells

**Fig. 7:**
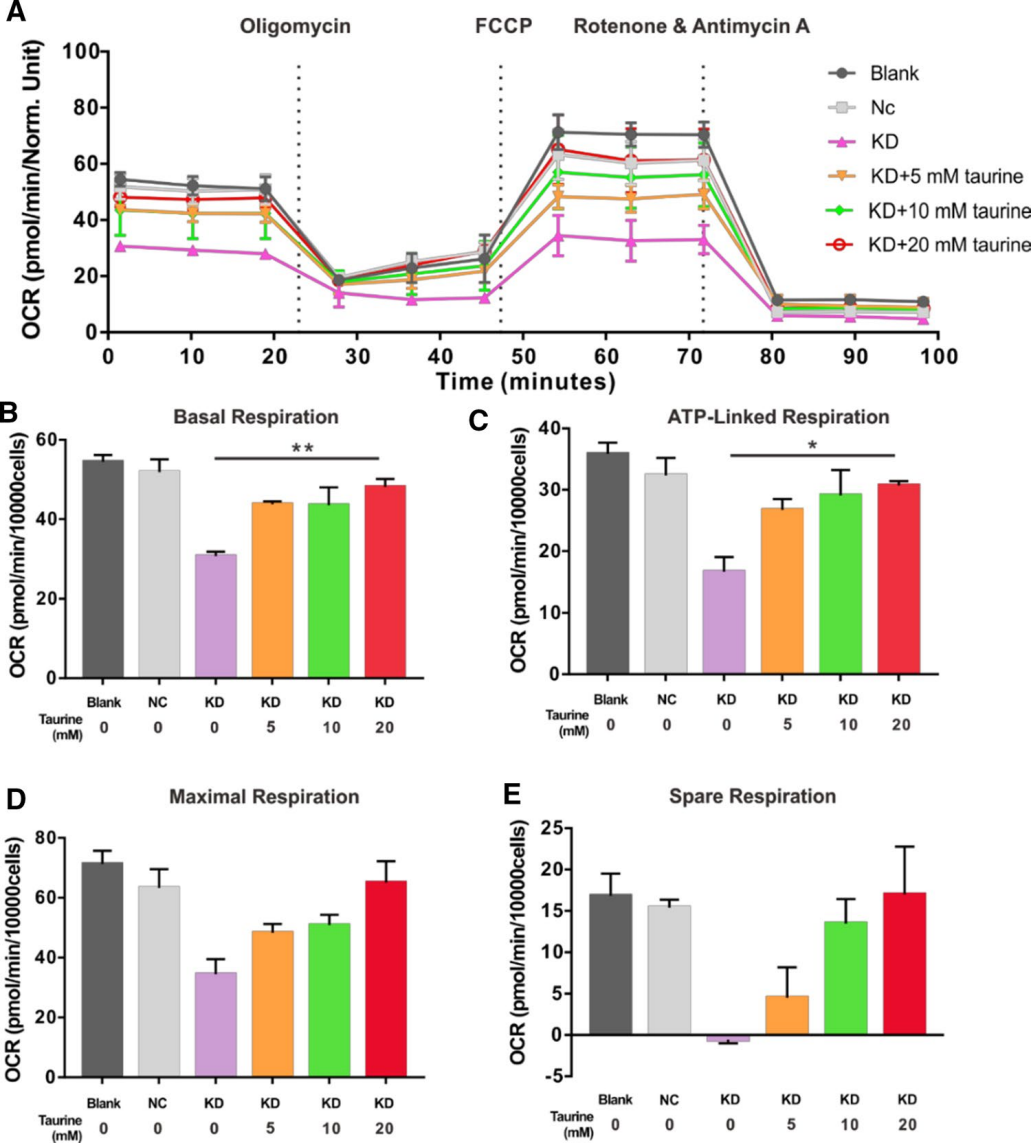
High concentrations of taurine improve the mitochondrial respiration of GTPBP3 knocked down R28 cells. **A** OCR was recorded at baseline and after the sequential injection of the three drugs, **B**–**E** The analysis of basal respiration, ATP-linked respiration, maximal respiration and spare respiratory capacity. *p < 0.05, **p < 0.01

Because the basal level of GTPBP3 was decreased in the KD group, we suspect whether there is a greater need for taurine to exert a protective effect. First, we evaluated the effects of high taurine concentrations on R28 and KD cells under normal condition. Treatment with 10 and 20 mM taurine for 24 h had no significant effect on cell viability but reduced the cytotoxicity of R28 cells and KD cells (Supplementary Fig. 1A–E). Moreover, the MMP of R28 cells could be increased by both 10 and 20 mM taurine, and the MMP of KD cells could only be increased by 20 mM taurine (Supplementary Fig. 1C, F). We also found that 10 and 20 mM exogenous taurine significantly increased intracellular taurine levels under stressful conditions (Supplementary Fig. 2). Next, we administered 10 and 20 mM taurine to the KD group under H/R injury and found the viability, cytotoxicity, MMP, and ROS levels were reversed. We also evaluated mitochondrial respiration in the KD group treated with high taurine concentrations. As shown in Fig. 7, 10 and 20 mM taurine both enhanced basal respiration, ATP-linked OCR, maximal respiration and spare respiratory capacity in the KD group. These results indicate that the effects of taurine in counteracting the effects of decreased GTPBP3 levels are dose-dependent, although the underlying mechanisms remain to be elucidated.

## Discussion

This study demonstrated that taurine can protect R28 cells under glaucomatous injury in vitro (oxidative stress induced by H_2_O_2_ and H/R-induced damage, which mimics the ischemia/reperfusion condition in glaucoma (Kim and Byzova 2014)) and explored a new mechanism of the neuroprotective effects of taurine, which depend on the GTPBP3-mediated taurine modification of mt-tRNAs and the promotion of mitochondrial energy metabolism.

In our study, we used immortalized R28 cells derived from postnatal day six rat retinal cells. This cell line is a retinal neural precursor cell line and express abundant retinal and neuronal markers, including the RGC marker RBPMS (Supplementary Fig. 3). It also possesses the functional capacity to respond to specific neurotransmitters, such as dopamine, acetylcholine, serotonin, and glycine (Seigel et al. 2004). R28 cells have been used to study in vitro toxicity and neuroprotection, gene expression, cell signaling pathways and retinal function. (Seigel 2014). Because proliferative R28 cells retain functional neuronal properties and can be propagated indefinitely, they are a promising research tool for in vitro studies of neuronal activity.

Taurine is the most abundant amino acid in the retina (Ripps and Shen 2012). R28 cells have a basal level of taurine, which can be increased by exogenous taurine treatment in a dose-dependent manner, indicating that exogenous taurine treatments have the potential to change intracellular activities by influencing intracellular taurine level. Although taurine was initially thought to be involved in photoreceptor degeneration, studies have shown that taurine depletion is also involved in retinal diseases with RGC degeneration, such as glaucoma and diabetic retinopathy (Froger et al. 2014). Taurine transporter-null mice display severe retinal degeneration. (Baliou et al. 2020; Heller-Stilb et al. 2002). A systematic review revealed that taurine and hypo-taurine metabolism was significantly enriched in the aqueous humor of patients with open angle glaucoma patients (Wang et al. 2021). These results demonstrated that taurine is crucial for the development of retinal degeneration. The most recognized property of taurine is oxidative resistance. However, some researchers have demonstrated that taurine cannot directly eliminate oxidants (Aruoma et al. 1988). The mechanisms underlying the protection effects of taurine on the retina remain to be elucidated.

Taurine participates in the modifications of five mt-tRNAs: τm^5^U in mt-tRNA^Leu(UUR)^ and mt-tRNA^Trp^, τm^5^s^2^U in mt-tRNA^Lys^, mt-tRNA^Glu^, and mt-tRNA^Gln^. This taurine-linked, post-transcriptional modification of the wobble base enhances the binding of the anticodon and codon and improve the translation of 13 subunits of the ORC. Once the synthesis of mitochondria-encoded subunits declines, the integrity of the electron transport chain (ETC) is reduced, followed by reduced activities of the five ETC complexes (Ricci et al. 2008). The decreased activity of the complexes slows the electron flux through the respiratory chain and results in electrons diverting from complexes I and III to alternate acceptors, such as oxygen, from which superoxide is generated (Balaban et al. 2005). Because of the importance of taurine in mt-tRNA modification and the promotion of the function of the ETC, along with the result observed in our study that taurine could promote mitochondrial energy metabolism, we propose that the antioxidant property of taurine in R28 cells is based on reducing the production of oxidants rather than eliminating them. This theory was also demonstrated for cardiomyocytes (Jong et al. 2012).

GTPBP3 is the key enzyme involved in taurine modification, and its deficiency is associated with a combined oxidative phosphorylation deficiency (Kohda et al. 2016). Partial inactivation of GTPBP3 in zebrafish embryos affects the development of various organs, including the eyes (Chen et al. 2019, 2016). The results of whole exome sequencing suggested that GTPBP3 might be related to the development of glaucoma (Qiao et al. 2020). In our study, we discovered for the first time that taurine regulates the expression of GTPBP3 in R28 cells under different conditions. By knocking down GTPBP3 in R28 cells, we revealed that the decreased GTPBP3 level substantially influenced the proliferation rate which could not be enhanced until 72 h after taurine treatment. The mechanism for this result may be that the reduced GTPBP3 level takes more time than normal basal GTPBP3 level to be up-regulated by taurine.

Moreover, mitochondrial energy metabolism, especially spare respiratory capacity was influenced by decreased GTPBP3 level. Spare respiratory capacity is defined as the difference between the ATP produced by oxidative phosphorylation at basal and maximal activity, which reflects the ability of cells to manage various stresses or increased workloads. Cells with deficit spare respiratory capacity are more likely to die than normal cells. In our study, the protective effects of low doses of taurine disappeared in the KD group. Although 5 mM taurine seemed to promote OCR in the KD group, the spare respiratory capacity did not recover. These results demonstrated that GTPBP3-mediated taurine modification is of substantial importance for taurine protection.

Notably, we also observed that relatively high concentrations of taurine had protective effects on KD cells. Jong et al., stated that the mitochondrial content of taurine was ~ 30% of total cellular levels (Jong et al. 2010). Mitochondria are where taurine modification of mt-tRNAs occurs, suggesting that the mitochondrial pool of taurine is important in modification. Although we did not measure mitochondria taurine concentration, there is indeed a possibility that mitochondria taurine concentration increased with increasing intracellular taurine concentration, which may be one of the explanations that only high concentrations of taurine are effective in reversing the effects of the KD group. Another possibility is that the reduction of GTPBP3 is more severe in KD cells than that of R28 cells under damages, and KD cells requires more taurine than R28 cells to promote GTPBP3 for improving cell physiological function. Other signaling pathways may be involved in the taurine-mediated regulation of GTPBP3 and damage caused by GTPBP3 reduction. Fakruddin et al. reported that defective mt-tRNA taurine modification not only leads to mitochondrial dysfunction but also activates endoplasmic reticulum (ER) stress and unfolded protein response (UPR), which have profound impacts on cellular proteostasis and homeostasis (Fakruddin et al. 2018). A chemical chaperone, taurine-conjugated bile acid, tauroursodeoxycholate (TUDC), can suppress the UPR and reduce cytotoxicity induced by defective mt-tRNA taurine modification. Studies have shown that ER stress and oxidative stress are closely interrelated and can potentially cause a vicious cycle of damage (Malhotra and Kaufman 2007). The action of taurine on ER stress in stroke models has been described (Jakaria et al. 2019; Gharibani et al. 2013). The UPR is involved in many ocular disorders, such as glaucoma, optic neuropathies, and retinal degeneration diseases (Boriushkin et al. 2015; Kroeger et al. 2019). Whether the UPR is involved in the regulation of GTPBP3 by taurine in retinal cells remains to be explored in our next work.

This study has some limitations. Although the R28 cell line is a promising, convenient tool for retinal-related in vitro studies, it cannot completely replace primary retinal cells that mimic the in vivo state and physiology. In this study, we explored only the protective effects of taurine in vitro*.* An in vivo study exploring the effects of taurine in mice with ischemia/reperfusion injury is necessary to further clarify the protective role of taurine in glaucoma. Furthermore, the modification level of mt-tRNAs and ETC complex activity under different conditions is worth detecting, which would provide additional direct evidence that reveals the mechanisms of taurine’s protective effects.

## Conclusion

In summary, our study explored a new mechanism of the neuroprotective effects of taurine, which depend on the GTPBP3-mediated taurine modification of mt-tRNAs and the promotion of mitochondrial energy metabolism. Interventions that modulate molecules related to this mechanism can be used as potential novel therapeutic targets.

## Supplementary Information

Below is the link to the electronic supplementary material.

Supplementary file1 (DOCX 5245 KB)
