# Supplementary material for: Taurine protects R28 cells from hypoxia/re-oxygenation-induced damage via regulation of mitochondrial energy metabolism
Source: Amino Acids. 2022 Sep 2;54(12):1585–99. doi: 10.1007/s00726-022-03199-5 (PMC9708803; doi:10.1007/s00726-022-03199-5)
Supplement: Supplementary file 1 — Supplementary file1 (DOCX 5245 KB) [file 726_2022_3199_MOESM1_ESM.docx]

**Taurine protects R28 cells from hypoxia/reoxygenation-induced damage via regulation of mitochondrial energy metabolism**

Wei Lu^a^, Yuting Yang^a^, Shunxiang Gao^a^, Jihong Wu^a^, Xinghuai Sun^a, b, c*^

^a^Department of Ophthalmology & Visual Science, Eye & ENT Hospital, Shanghai Medical College, Fudan University, Shanghai 200031, China

^b^State Key Laboratory of Medical Neurobiology and MOE Frontiers Center for Brain Science, Institutes of Brain Science, Fudan University, Shanghai 200032, China

^c^NHC Key Laboratory of Myopia, Chinese Academy of Medical Sciences, and Shanghai Key Laboratory of Visual Impairment and Restoration (Fudan University), Shanghai 200031,

*** Correspondence:**Xinghuai Sun
xhsun@shmu.edu.cn

Journal Name: **Amino Acids**


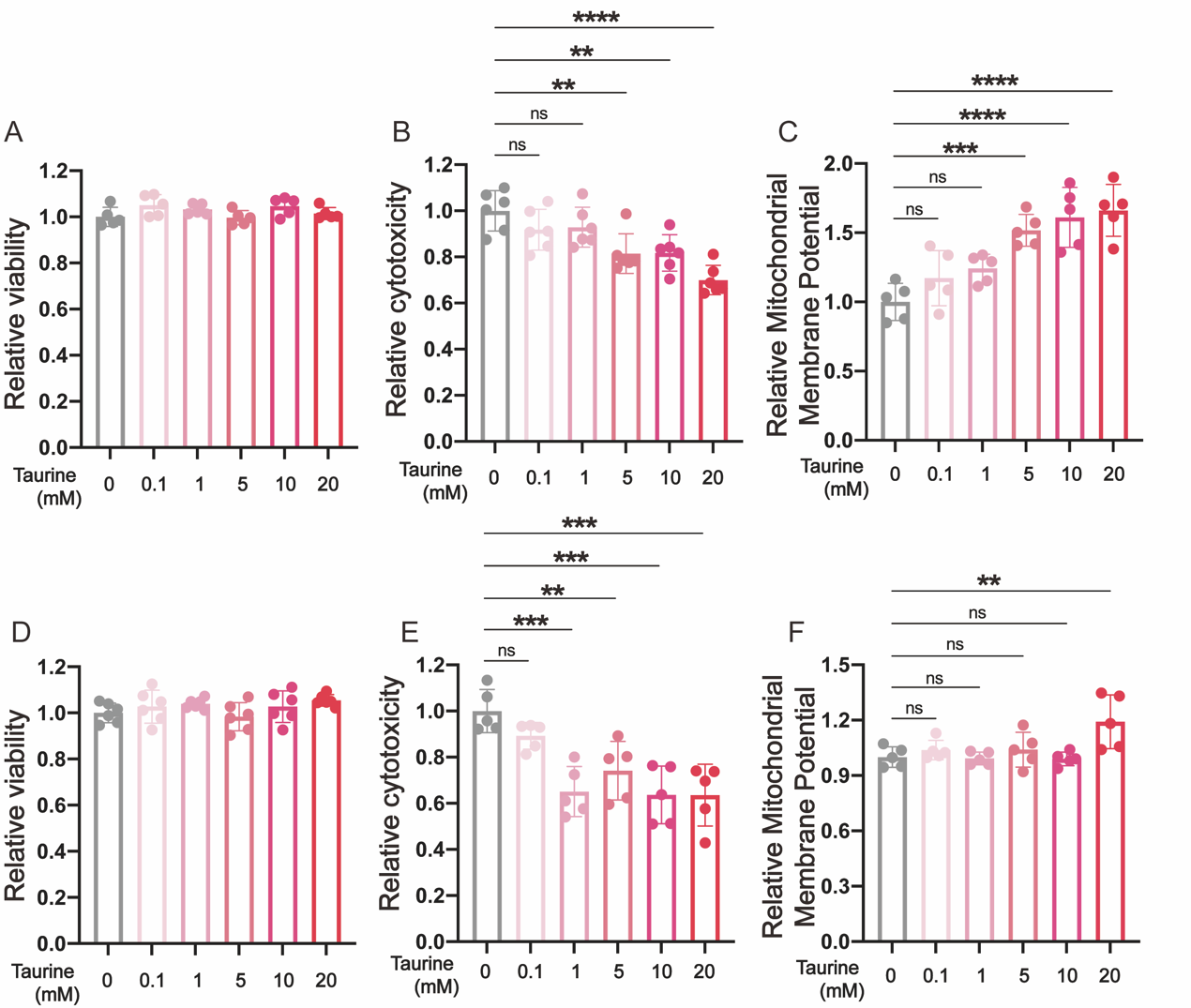


Supplementary Fig. 1 (A-C) CCK8, LDH and MMP level of R28 cells treated with different concentrations of taurine. (D-F) CCK8, LDH and MMP level of KD cells treated with different concentrations of taurine. All results were reported as mean ± standard deviation (SD). **p<0.01, ***p<0.001, ****p<0.0001.


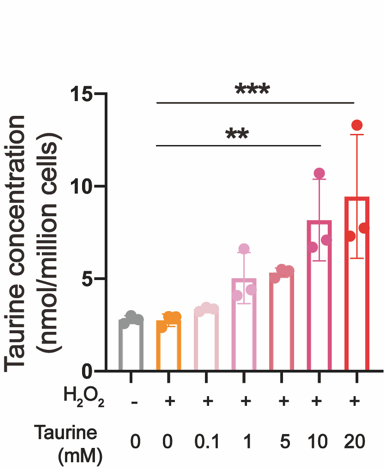


Supplementary Fig. 2 Taurine contents in R28 cells during H_2_O_2_-induced oxidative stress. All results were reported as mean ± SD. **p<0.01, ***p<0.001.


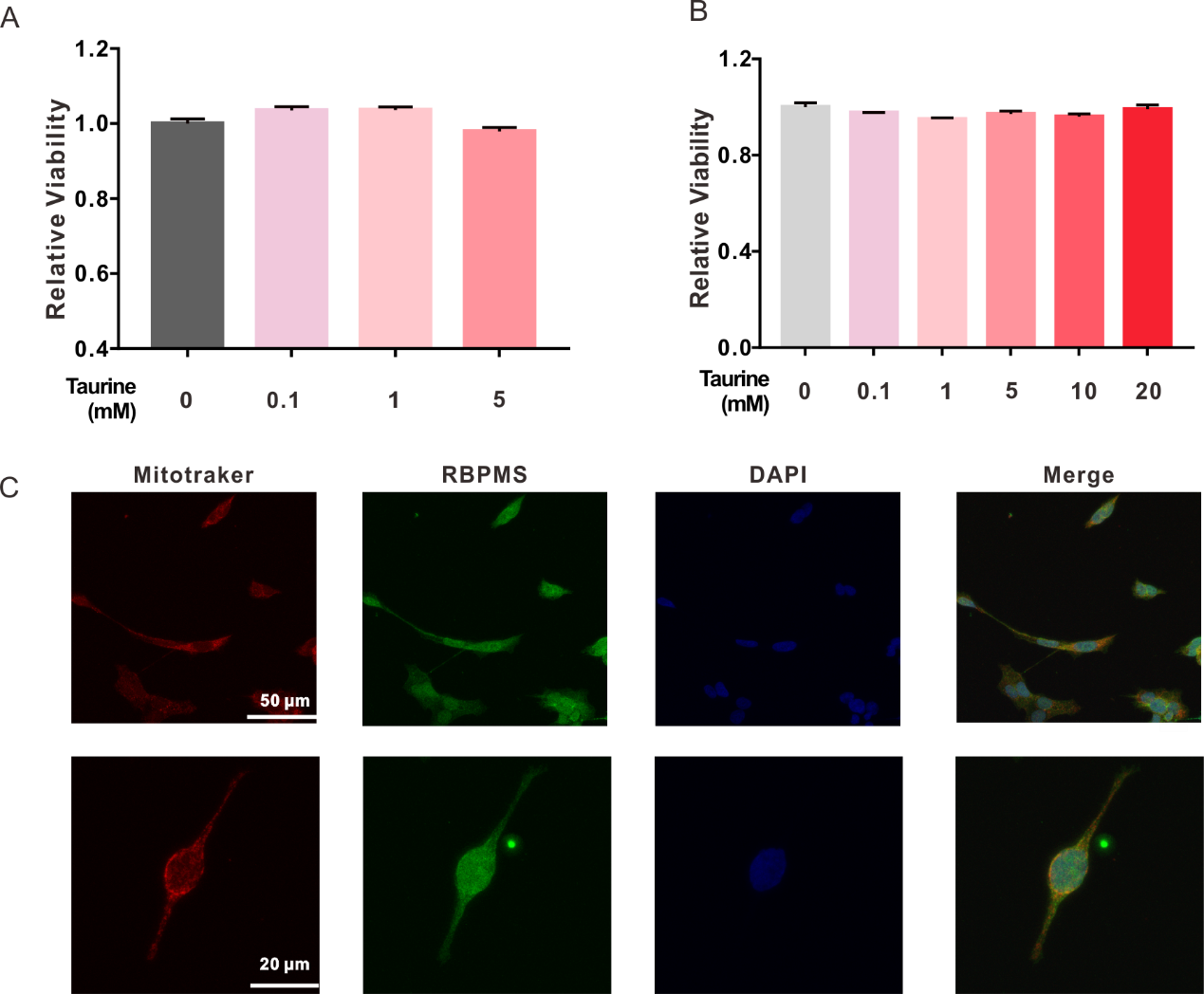


Supplementary Fig. 3 The representative images of the staining of mito-tracker and RBPMS in R28 cells
